# Evaluation of genetic diversity among Russet potato clones and varieties from breeding programs across the United States

**DOI:** 10.1371/journal.pone.0201415

**Published:** 2018-08-01

**Authors:** Sapinder Bali, Girijesh Patel, Rich Novy, Kelly Vining, Chuck Brown, David Holm, Gregory Porter, Jeffrey Endelman, Asunta Thompson, Vidyasagar Sathuvalli

**Affiliations:** 1 Hermiston Agricultural Research and Extension Center, Oregon State University, Hermiston, Oregon, United States of America; 2 Department of Oncological Studies, Mitchell Cancer Institute, University of South Alabama, Mobile, Alabama, United States of America; 3 United States Department of Agriculture, Agricultural Research Service, Aberdeen, Idaho, United States of America; 4 Department of Horticulture, Oregon State University, Corvallis, Oregon, United States of America; 5 United States Department of Agriculture, Agricultural Research Service, Prosser, Washington, United States of America; 6 Department of Horticulture and Landscape Architecture, San Luis Valley Research Center, Colorado State University, San Luis, Colorado, United States of America; 7 School of Food and Agriculture, University of Maine, Orono, Maine, United States of America; 8 Department of Horticulture, College of Agricultural and Life Sciences, University of Wisconsin, Madison, Wisconsin, United States of America; 9 Department of Plant Sciences, North Dakota State University, Fargo, North Dakota, United States of America; 10 Department of Crop and Soil Science, Oregon State University, Corvallis, Oregon, United States of America; National Cheng Kung University, TAIWAN

## Abstract

DNA fingerprinting is a powerful tool for plant diversity studies, cultivar identification, and germplasm conservation and management. In breeding programs, fingerprinting and diversity analysis provide an insight into the extent of genetic variability available in the breeding material, which in turn helps breeders to maintain a pool of highly diverse genotypes by avoiding the selection of closely related parents. Oblong-long tubers with russeting skin characterize Russet potato, a primary potato market class in the United States, and especially in the western production regions. The aim of this study was to estimate the level of genetic diversity within this market class potato, utilizing clones and varieties from various breeding programs across the United States. A collection of 264 Russet and non-Russet breeding clones and varieties was fingerprinted using 23 highly polymorphic genome-wide simple sequence repeat (SSR) markers, resulting in 142 polymorphic alleles. The number of alleles produced per SSR varied from 2 to 10, with an average of 6.2 alleles per marker. The polymorphic information content and expected heterozygosity of SSRs ranged from 0.37 to 0.89 and 0.50 to 0.89 with an average of 0.77 and 0.81, respectively. Out of these 23 markers, we propose nine SSR markers best suited for fingerprinting Russet potatoes based on polymorphic information content, heterozygosity and ease of scoring. Diversity analysis of these clones suggest that there is significant diversity across the breeding material and the diversity has been evenly distributed among all the regional breeding programs.

## Introduction

Potato (*Solanum tuberosum* L.) is the third most important carbohydrate source in the human diet after rice and wheat [1]. It is an autotetraploid, vegetatively propagated crop grown in temperate, subtropical and tropical regions [2, 3]. *Solanum* is one of the most species-rich genera of flowering plants [4], and potato possesses tremendous intraspecies genetic variability and morphological plasticity. The ploidy level varies from diploid to hexaploid [5]; however, the cultivated potato is generally tetraploid (2n = 4x = 48). In addition to being consumed fresh and in various processed forms, potato has important industrial applications that includes manufacturing starch, processed foods and alcoholic beverages. Potato tubers contain valuable nutrients including carbohydrates, vitamins, proteins, fiber, antioxidants, calcium, potassium, phosphorus and iron [6–8].

Among various potato types grown in the United States, Russet potato is the most popular market class. Russet potatoes are oval-oblong to long with russeting skin that varies in color from tan to darker brown and is characterized by netting on the skin. The flesh is usually white and very firm. Russet potato tubers usually range from 3–8 inches in length and 1.5–3 inches in width (76.2–203.2mm length and 38.1–76.2mm width). Russet potatoes in the United States are consumed mostly as French-fries, baked, mashed, roasted or dehydrated. The higher dry matter content of this market class is desirable for low oil uptake during frying, thus making Russet potatoes one of the best choice for French-fries [9].

The evaluation and quantification of genetic variation in plants is an important aspect of breeding and crop improvement programs. Molecular markers are important tools in plant breeding programs that can be utilized to improve yield, quality, disease resistance and stress tolerance [10]. Genetic markers based on simple sequence repeats (SSRs) are highly polymorphic, co-dominant, well conserved across related species and follow Mendelian inheritance patterns, which makes them ideal markers for studying genetic diversity [11–13]. SSRs have been extensively used in diversity analysis and identification of cultivated potato clones [14–24]. Although, many SSR studies have been done in potato in general, SSR analysis within the Russet group is very limited. The only study done on Russet varieties to date is by Karaagac et al. [25], who used 25 SSR markers to genotype 54 potato clones including some released Russet varieties and found that all the Russet clones fell within the same cluster. With increasing interest in the processing products of potatoes worldwide, Russet varieties being the primary market class for this end use, it is important to understand the diversity within the Russet potato gene pool utilized among the breeding programs throughout the United States. The major objective of the present study is to quantify the genetic variability among the Russet potato clones and study the genetic relationships based on their pedigrees. This study will also determine whether the available markers can be utilized to distinguish among Russet breeding clones currently being evaluated by breeding programs for release as improved potato varieties.

## Materials and methods

### Plant materials

Tubers from 264 potato clones were collected from seven major potato-breeding programs across the United States [Pacific Northwest (NWPVD), Colorado (CO), Maine (ME), Minnesota (MN), Wisconsin (WI), North Dakota (ND) and Maryland (MD)]. This collection includes 198 Russet breeding selections, 50 released Russet varieties and 16 non-Russet potatoes (chip, specialty and germplasm). The details of Russet selections, including pedigree information and the associated breeding programs is presented in the S1 Table. The details of released Russet varieties and non-Russet potato clones used in the study are presented in Table 1 and Table 2, respectively. Seed tuber pieces were planted in the greenhouse and leaf material was collected from the emerged shoots for DNA isolation.

**Table 1:** List of 50 released clones (Russet type) used in the present study.

Breeding program	Sample Name	Female parent	Male parent
Northwest Potato Variety Development Program (NWPVD)	Alpine Russet	A8343-12	A85103-3
	Blazer Russet	A7816	Norking Russet
	Castle Russet	PA00V6-3	PA01N22-2
	Century Russet	A6789-7	A6680-5
	Classic Russet	Blazer Russet	Summit Russet
	Clearwater Russet	Bannock Russet	A89152-4
	Defender	Ranger Russet	KSA195-90
	Echo Russet	A89222-3	COA90064-6
	Gem Russet	A77182-1	Russet Norkotah
	GemStar Russet	Gem Russet	A8341-5
	Highland Russet	Ranger Russet	Russet Legend
	Klamath Russet	A79172-6	Russet Norkotah
	Owyhee Russet	A89384-10	A89512-3
	Pallisade Russet	AWN86514-2	A86102-6
	Payette Russet	EGAO9702-2	GemStar Russet
	Pioneer Russet	A7816-14	Russet Norkotah
	Premier Russet	A87149-4	A88108-7
	Ranger Russet	Butte	A6595-3
	Sage Russet	A89384-10	A91194-4
	Summit Russet	A77236-6	TND329-1Russ
	Targhee Russet	A92303-7	A96004-8
	Teton Russet	Classic Russet	Blazer Russet
	Umatilla Russet	Butte	A77268-4
	Wallowa Russet	Ranger Russet	A82758-3
	Western Russet	A68113-4	Belrus
Colorado State University—San Luis Valley Research Center, Center, Colorado (CO)	Canela Russet	A8343-12	A8784-3
	Centennial Russet	W12-3	Nooksack
	Keystone Russet	A76147	A7875-5
	Mesa Russet	AO80432-1	Silverton Russet
	Rio Grande Russet	Butte	A8469-5
	Russet Nugget	Krantz	AND71609-1
	Silverton Russet	CalWhite	A7875-5
	Ute Russet	W12-3	Nooksack
	Crestone Russet	AC91014-2	Silverton Russet
	Mercury Russet	AC93047-1	Silverton Russet
	Fortress Russet	AWN86514-2	A89384-10
North Dakota State University, Fargo, North Dakota (ND)	Dakota Russet	Marcy	AH66-4
	Dakota Trailblazer	A98163-3LS	A8914-4
	Russet Norkotah	ND9687-5 Russ	ND9526-Russ
	Russet Norkotah-S3	ND9687-5 Russ	ND9526-Russ
	Russet Norkotah-S8	ND9687-5 Russ	ND9526-Russ
University of Maine, Orono, Maine (ME)	Allagash Russet	BR7093-56	B6024-3
	Reeves Kingpin	CS7981-7	CF7608-19
	Caribou Russet	Reeves Kingpin	Silverton Russet
USDA/ARS, Beltsville, Maryland (MD)	Coastal Russet	Russet Burbank	B8281-5
	Belrus	W245-2	Penobscot
University of Wisconsin, Madison, Wisconsin (WI)	Freedom Russet	ND14-1	W1005rus
	Millennium Russet	Atlantic	FL1154rus
BV de ZPC, Netherlands	Innovator	Shepody	RZ-84-2580
Unknown	Russet Burbank	Early Rose	??

**Table 2:** List of 16 non-Russet clones used in the present study.

Breeding program	Sample Name	Female parent	Male parent
Northwest Potato Variety Development Program (NWPVD)	TerraRossa	PA97B35-2	PA97B29-3
	Yukon Nugget	PA99P35-1	Rose Gold
	A00ETB12-3	A92303-7	ETB6-21-3
	P2-4	2-7-4D	Katahdin
Colorado State University—San Luis Valley Research Center, Center, Colorado (CO)	Chipeta	WNC612-13	Wischip
	Masquerade	Inca Gold	A91846-5R
	Harvest Moon	Inca Gold	A89655-5DY
International Potato Center (CIP), Lima, Peru	LBR-8	387348.2	390357.4
	Tacna	720087	386287–1
USDA/ARS, Beltsville, Maryland (MD)	Atlantic	Wauseon	Lenape
University of Wisconsin, Madison, Wisconsin (WI)	Snowden	Lenape	Wischip
Louisiana State University, Baton Rouge, Louisiana (LA)	Red La Soda	Triump	Katahdin
Cornell University, Itaca, New York (NY)	Q115-6 PTW	L 227	790–82
Agriculture Canada and University of Guelph, Ontario, Canada	Yukon Gold	Norgleam	W5279-4
Agriculture Canada, New Brunswick, Canada	Shepody	F58050	Bake King
Common Wealth Potato Collection, Scotland, United Kingdom	Pallida CPC (12764AB1)	?	?

### Genomic DNA extraction

DNA was isolated from young tender leaves using Mag-Bind^®^ Plant DNA plus 96 Kit (Omega Bio-tek, Norcross, Georgia, USA) according to the instruction manual. DNA quality and quantity was determined by agarose gel electrophoresis and spectrophotometer (Nano Drop™, Thermo Scientific, Waltham, Massachusetts, USA) respectively. All the samples were diluted to 20 ng/μl concentration with nuclease-free water.

### SSR fingerprinting

#### Primer testing

Thirty-two SSR markers identified previously as being highly informative in potato [17, 21, 24, 26–28] [Scottish Crop Research Institute (SCRI, unpublished)], were selected and used on 24 Russet potato clones to identify primer pairs with scorable polymorphisms. Twenty-three primer pairs were shortlisted and used to fingerprint 264 potato clones for further analysis (Table 1, Table 2, and S1 Table). The majority of these SSR markers (60%) are composed of trinucleotide repeat motifs, followed by 30% di and 10% tetra nucleotide repeat motifs. The forward primer of SSR marker was fluorescently labeled with either 6-FAM, 5-HEX or NED. PCR products of 8–9 primer pairs each with three different fluorescent label and compatible amplicon sizes were multiplexed before capillary electrophoresis.

#### Polymerase chain reaction

Polymerase chain reactions (PCR) were performed in 10 μl volumes using 1X AmpliTaq Gold® 360 master mix (Life Technology, Carlsbad, California, USA), 0.2 μM of each primer (forward and reverse) and 20 ng DNA. The amplification cycle was performed on a 96 well Thermal cycler (Applied Biosystems, Foster City, California, USA) as follows: one cycle at 95°C for 5 min followed by 40 cycles at 95°C for 40 s, annealing at 54–60°C for 50 s, 72°C for 40 s, ending with one cycle at 72°C for 10 min. PCR amplicons were separated on 2% agarose gel with 100 bp DNA ladder (Promega, Madison, Wisconsin, USA) as size standard. Gels were stained with ethidium bromide (0.5 μg/ml) for 20 minutes and de-stained with distilled water for 20 min on an orbital shaker. DNA bands were visualized and recorded on GelDoc™ XR+ (Bio-Rad, Hercules, California, USA).

#### Capillary electrophoresis

Two microliters of labeled PCR product from each primer pair were pooled to prepare respective multiplex set and diluted with sterile deionized water up to a final volume of 180 μl. Subsequently, 1.2 μl aliquot of the diluted sample was denatured and size fractioned using capillary electrophoresis on an ABI 3730 DNA analyzer (Applied Biosystems, Life Technologies, Carlsbad, California, USA) with an internal-lane size standard (GeneScan^TM^ 500 ROX^TM^) at core facility of the Center for Genomic Research and Biocomputing (CGRB), Oregon State University, Corvallis, Oregon, USA.

### Data analysis

#### SSR genotyping, Neighbor-Joining and STRUCTURE analysis

Capillary electrophoresis data was scored using GeneMapper® Software v4.1. (Applied Biosystems, Foster City, California, USA). Peak sizes were recorded and the number of alleles, polymorphic information content (PIC) and expected heterozygosity (H_e_) were calculated using an online PIC calculator (https://www.liverpool.ac.uk/~kempsj/pic.html). SSR fingerprints of 264 potato clones is presented in S1 File. Binary data (0/1) was used to calculate a “dissimilarity index” using Jaccard coefficient. Factorial analysis was performed using dissimilarity index and a genetic diversity tree (dendrogram) was constructed using the weighted Neighbor-Joining (NJ) method in Darwin 6.0.1.2 [29]. Genetic structure analysis was performed using Bayesian method based interactive software, Structure 2.3.4 [30]. Based on the results of NJ analysis, the hypothetical number of sub-populations (K = 1 to 10) was run at three independent replicates at Burnin period length of 100,000 and 200,000 Markov Chain Monte Carlo (MCMC). The value of ΔK was calculated using Evanno’s method in Structure Harvester [31, 32]. In order to determine the population stratification, 264 clones were run at K = 3 with a Burnin period length of 100,000 and 200,000 MCMC using admixture model. Allele frequency divergence among the clusters, fixation index (F_st_) and the average distance among individuals in the same cluster was calculated using Structure 2.3.4.

#### SNP genotyping

In order to measure the differentiation power of SSR markers, SSR data of a subset of 21 Russet clones was compared with SNP data generated in our previous study [33]. Briefly, SNP genotyping was performed using Infinium SolCAP 12K array (12,808 SNPs) and intensity data was analyzed using GenomeStudio (Illumina, San Diego, California, USA). SNP markers with ≤ 10% “no call” rate were dropped from the study. NJ trees were constructed using SSR and SNP marker data separately in Darwin 6.0.1.2 as described above. A tanglegram [34] comparing the NJ trees was constructed using Dendroscope 3.5.9 [35].

## Results and discussion

### SSR fingerprinting

Of 32 SSR markers tested on a set of 24 Russet clones, 25 markers showed scorable polymorphism. Two of the markers (STM1106 and STI0003) were dropped from the final analysis, as they produced inconsistent allelic patterns. In total, 23 markers that produced clear consistent polymorphic alleles (Table 3) were used for genotyping 264 potato clones collected from various potato breeding programs (Table 1, Table **2** and S1 Table). For multiplexing, ease and accuracy of scoring alleles, primers were fluorescently labeled and separated on capillary electrophoresis (Table 3). Twenty-three SSR markers used in the study spanned all 12 chromosomes of the potato genome with an average of two markers per chromosome. Chromosome VIII had a maximum of five markers whereas, Chromosome V and X had one marker each. The observed product size and the expected product size showed minor variations in a few of the markers but the majority of them were within the expected range (Table 3). The total number of alleles ranged from 2–10 with an average of 6.2 alleles per marker. SSR marker STG0001 produced the maximum of 10 alleles while, STM1053 amplified only two alleles. Eleven of the markers produced rare alleles (alleles amplified only in upto two clones) (Table 3). Marker STM0030 produced a single rare allele of 125 bp in clone AO00710-1, whereas STI0023 amplified 178 bp allele in clone W8650-9. Marker STM5114 amplified a rare allele of 293 bp in two of the non-Russet clones ‘LBR-8’ and ‘Q115-6’. Marker STG0016 produced two rare alleles of 120 bp and 140 bp in AF4124-4, AF4124-7 and AO06822-2, A06866-2, respectively. Marker STM5140 amplified a single rare allele of 170 bp in clones AF4749-5 and CO00254-9, whereas STWAX-2 produced a 220 bp allele in AF3016-2 and AC99329-7. In addition, marker STG0004 amplified 190 bp allele in A07016-1TE, STM1064 amplified 184 bp allele in A07061-6, STI0004 amplified 89 bp allele in AF4953-6 and STG0001 amplified 133 bp allele in AF3016-2 clone. Marker STM1052 amplified 220 bp allele in A06014-14TE and 223 bp allele in AF4677-1 and AF4880-1. Marker STI0012 also produced two rare alleles, 153 bp and 172 bp alleles in A07061-6 and A08422-3 and AF465-2, respectively. Interestingly, marker STG0004 produced a 200 bp allele, which was specific to eight of the NWPVD clones namely, A06021-1T, A06084-1TE, A06130-3T, A06866-2, A07030-12TE, A07070-2, A07426-8LB and A08069-3 and STM1052 amplified a 212 bp allele specific to four NWPVD clones namely, A06130-3T, A06866-2, A07030-12TE and A07070-2. These two alleles were common in four of the clones namely, A06130-3T, A06866-2, A07030-12TE and A07070-2.

**Table 3:** Details of 23 SSR primers used in the present study.

Primer Name	Repeat Motif	Label	Reference	Chromosome location	Expected product size (bp)	Observed product size (bp)	Total number of alleles	Allele sizes observed (bp)	Unique alleles^1^	PIC	H_e_	Ease of Scoring^2^
STI0033	(AGG)n	FAM	Feingold et al. 2005	VII	109–130	109–130	6	109, 115, 122, 124, 128, 130	NA	0.8	0.83	1
STM1104	(TCT)n	FAM	Milbourne et al. 1998	VIII	164–198	162–173	5	162, 164, 167, 170, 173	NA	0.76	0.79	1
STM1016	(TCT)n	FAM	Milbourne et al. 1998	VIII	243–275	239–258	8	239, 241, 243, 246, 248, 251, 253, 258	NA	0.86	0.87	1
STM0030	(GT/GC)n	HEX	Milbourne et al. 1998	XII	109–163	125–166	9	125, 138, 140, 142, 146, 156, 160, 162,166	125 (AO00710-1)	0.87	0.88	3
STI0023	(CAG)n	HEX	Feingold et al. 2005	X	165–195	172–196	5	172, 175, 178, 193, 196	178 (W8650-9)	0.76	0.79	2
STM5114	(ACC)n	HEX	Scottish Crop Research Institute SCRI (unpublished)	II	286–295	281–299	7	281, 285, 288, 290, 293, 297, 299	293 (LBR-8/Q115-6)	0.86	0.87	1
STG0016	(AGA)n	NED	Ghislain et al. 2009	I	123–154	120–153	7	120, 123, 129, 132, 135, 140, 153	120 (AF4124-4/AF4124-7), 140 (AO06822-2/A06866-2)	0.83	0.85	1
STM5140	(AAT)n	NED	Scottish Crop Research Institute SCRI (unpublished)	IV	180–210	170–188	6	170, 173, 176, 179, 182, 188	170 (AF4749-5/CO00254-9)	0.81	0.83	1
STWAX-2	(ACTC)n	NED	Veilleux et al. 1995	VIII	220–235	220–238	8	220, 222, 225, 228, 230, 232, 234, 238	220 (AF3016-2/ AC99329-7)	0.86	0.87	3
STI0019	(ATCT)n	FAM	Feingold et al. 2005	VII	104–124	105–124	5	105, 109, 115, 119, 124	NA	0.76	0.79	2
STG0004	(GT)n	FAM	Ghislain et al. 2009	XI	212–217	190–200	6	190, 192, 194, 196, 198, 200	190 (A07016-1TE)	0.81	0.83	3
STI0030	(ATT)n	HEX	Feingold et al. 2005	XII	81–104	82–103	6	82, 85, 88, 91, 100, 103	NA	0.8	0.83	3
STG0010	(TG)n	HEX	Ghislain et al. 2009	III	159–166	159–168	4	159, 162, 166, 168	NA	0.7	0.74	1
STM1052	(AT)n GT(AT)n (GT)n	HEX	Milbourne et al. 1998	IX	207–224	207–250	8	207, 212, 217, 220, 223, 225, 228, 250	220 (A06014-14TE), 223 (AF4677-1/AF4880-1)	0.86	0.87	1
STI0038	(CTG)n	NED	Feingold et al. 2005	V	101–162	95–104	4	95, 98, 101, 104	NA	0.7	0.74	2
STM1064	(TA)n (TG)n GT (TG)N	NED	Milbourne et al. 1998	II	187–207	184–193	4	184, 187, 190, 193	184 (A07061-6)	0.7	0.74	2
STGBSS	(TCT)n	FAM	Provan et al. 1996	VIII	124–135	118–134	6	118, 121, 124, 128, 132, 134	NA	0.8	0.83	2
STM1053	(TA)n (ATC)n	FAM	Milbourne et al. 1998	III	167–196	168–170	2	168, 170	NA	0.37	0.5	1
STG0001	(CT)n	HEX	Ghislain et al. 2009	XI	125–163	123–143	10	123, 125, 127, 129, 131, 133, 135, 138, 141, 143	133 (AF3016-2)	0.89	0.89	1
STI0012	(ATT)n	HEX	Feingold et al. 2005	IV	166–185	153–188	9	153, 161, 164, 167, 170, 172, 182, 185, 188	153 (A07061-6/A08422-3), 172 (AF465-2)	0.87	0.88	1
STM5127	(TCT)n	HEX	Scottish Crop Research Institute SCRI (unpublished)	I	234–269	238–271	6	238, 241, 248, 250, 268, 271	NA	0.8	0.83	1
STI0014	(TGG)n (AGG)n	NED	Feingold et al. 2005	IX	118–157	118–131	5	118, 121, 125, 128, 131	NA	0.76	0.79	3
STI0004	(AAG)n	FAM	Feingold et al. 2005	VI	86–101	86–104	6	86, 89, 92, 97, 101, 104	89 (AF4953-6)	0.7	0.74	3

^1^ Clones associated with unique alleles are shown in the parenthesis

^2^ 1- Easy to score, 2- Moderate to score and 3- Hard to score.

Polymorphic information content (PIC) of 23 markers ranged from 0.37 (STM1053) to 0.89 (STG0001) with an average of 0.79 per marker whereas, the expected heterozygosity (H_e_) ranged from 0.50 (STM1053) to 0.89 (STG0001) with an average of 0.81 per marker. Similar values have been reported in fingerprinting a set of 40 Russet clones [33]. For future fingerprinting and genetic analyses, all the SSR markers in this study were rated on a scale of 1–3 for the ease of scoring (1- easy to score, 2-moderate and 3-difficult). The majority of the primers (12) were easy to score, followed by difficult to score (6) and moderate to score (5). We propose a set of best nine markers in terms of PIC, H_e_ scores and ease of scoring for Russet class potatoes. These markers include STI0033, STM1016, STM5114, STG0016, STM5140, STM1052, STG0001, STI0012 and STM5127. Four of these markers, STI0033, STM1016, STG0016 and STI0012 were also reported in our previous study [33] as the best markers for Russet potato varieties released by Northwest Potato Variety Development (NWPVD) based on their PIC, H_e_ values and ease of scoring.

### Genetic diversity analysis

**Fig 1:**
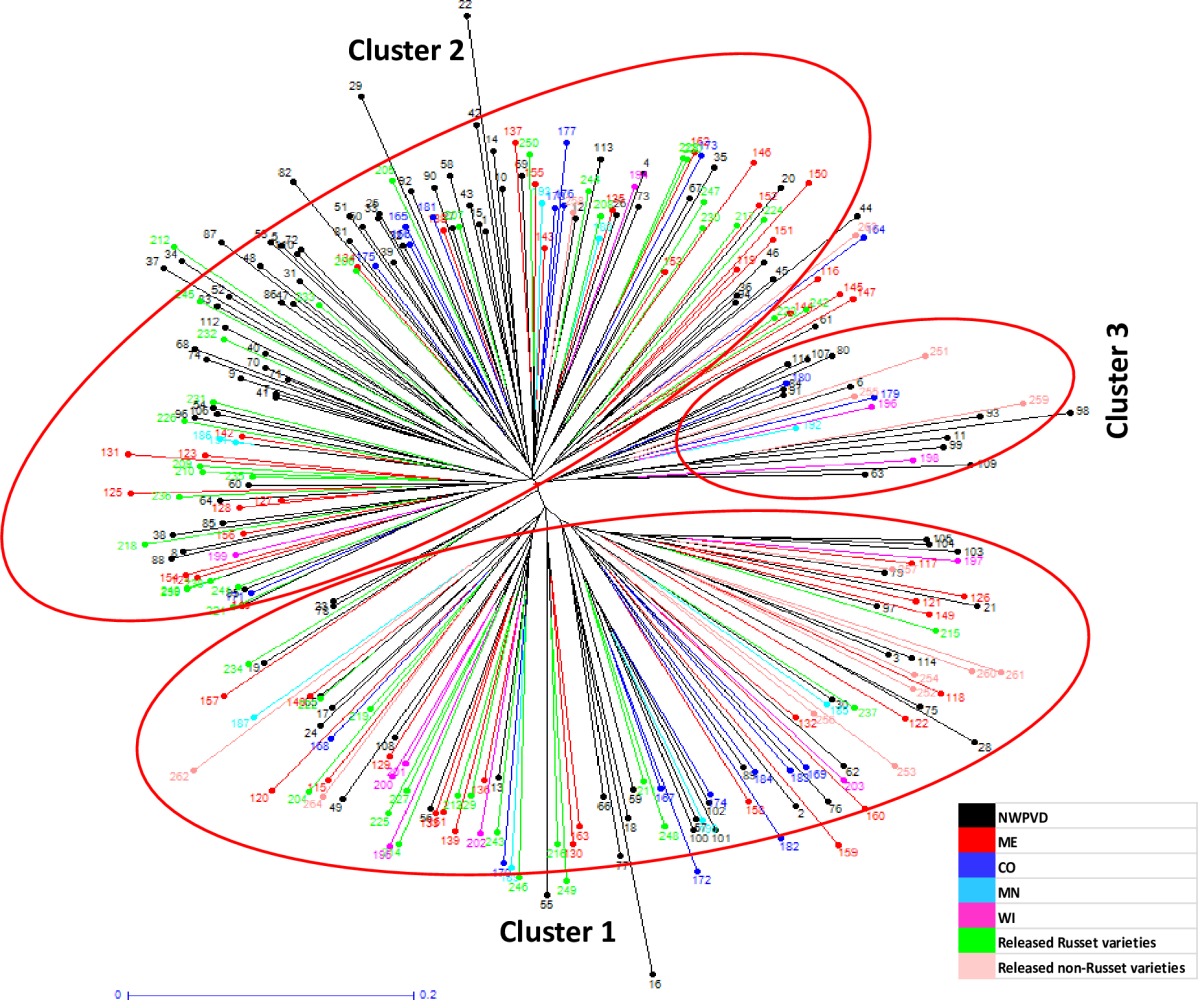
Unrooted neighbor-Joining tree of 264 clones (198 Russet selections, 50 released Russet varieties and 16 non-Russet clones) used in the present study. (CO: Colorado State University, NWPVD: Northwest Potato Variety Development Program, ME: University of Maine, MN: University of Minnesota, WI: University of Wisconsin) [Sample codes correspond to the data provided in S2, S3 and S4 Files].

Analysis of genetic diversity plays a major role in correct utilization of germplasm for crop improvement programs. Germplasm with high genetic variation is a valuable resource for breeding programs. The panel of 264 clones used in this study represent 198 Russet selections (NWPVD: 114, ME: 49, CO: 16, WI: 10 and MN: 09) (S1 Table), 50 released Russet varieties (NWPVD: 25, CO: 11, ND: 05, ME: 03, MD: 02, WI: 02, BV de ZPC, Netherlands: 01 and Unknown: 01) (Table 1) and 16 non-Russet clones (Table 2) from various potato breeding programs. Neighbor-Joining clustering analysis of these clones revealed three clusters (two major and one minor) (Fig 1). No tight clustering was observed based on the origin or geographical location of the Russet clones where they were bred, indicating adequate gene flow across the breeding programs. This is likely because of continuous exchange of early generation seedling tuber exchange among the potato breeding programs across the United States. However, groupings based on the lineage/pedigree of the clones were observed. As a result, Russet selections with one or both common parent tend to cluster together along with their parental clones in the same group (Group 1A, Group 1B, Group 2A, and Group 2B) (S2, S3 and S4 Files).

**Fig 2:**
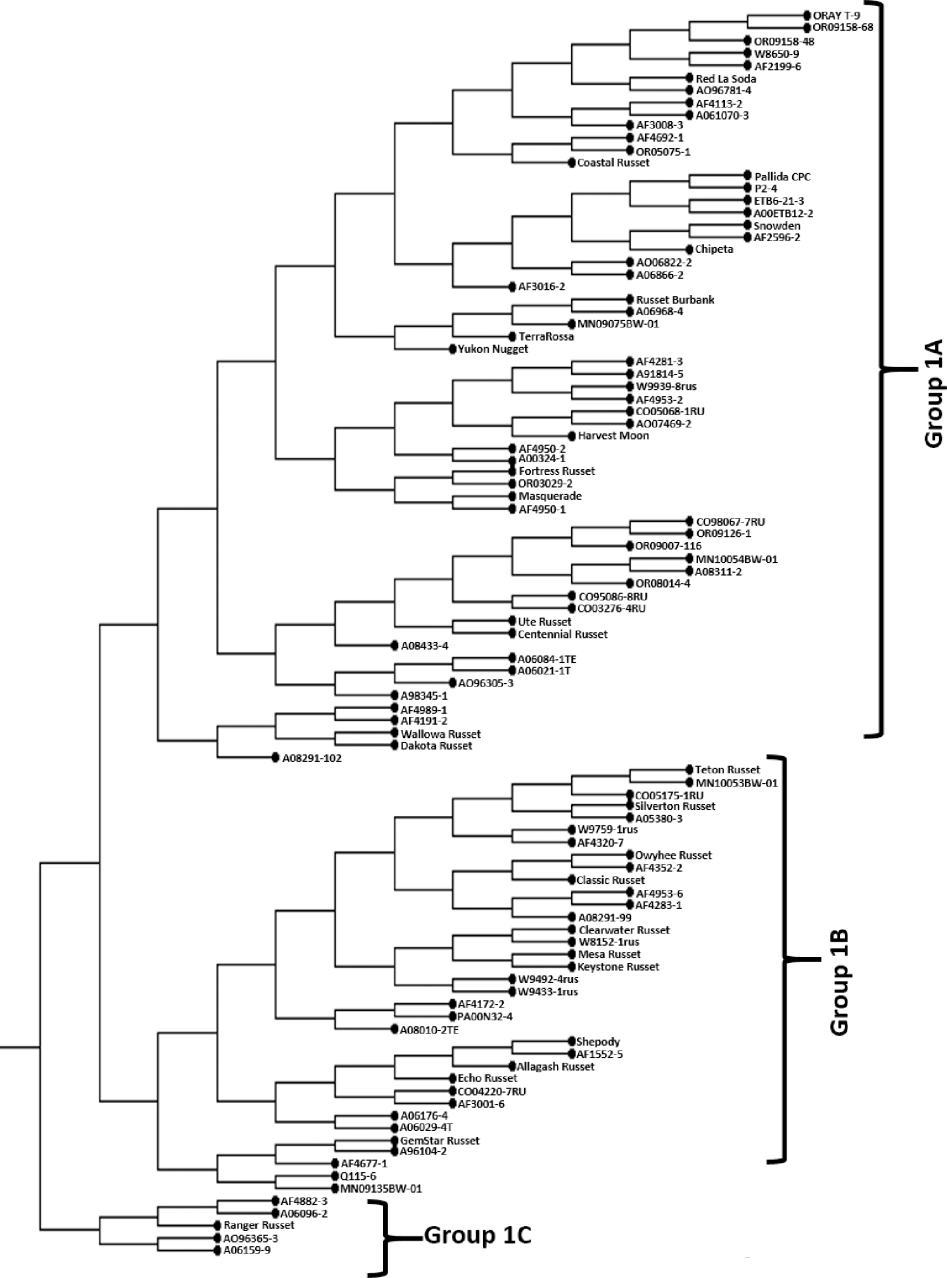
Detailed topology of Cluster 1.

Cluster 1 comprised of 101 clones and is further divided into three groups (1A, 1B and 1C) (Fig 2 and S2 File). Group 1A is the largest group in this cluster and is composed of a mix of Russet and non-Russet clones, 29 from NWPVD, 12 from ME, 10 from CO, three from WI, two from MN and one each from LA, MD, ND and Common Wealth Potato Collection, Scotland, United Kingdom. Nine out of 16 non-Russet clones, ‘Red La Soda’, ‘Pallida CPC’, P2-4’, ‘Snowden’, ‘Chipeta’, ‘TerraRossa’, ‘Yukon Nugget’, ‘Harvest Moon’ and ‘Masquerade’ are placed in this group along with promising Russet varieties, Coastal Russet, Fortress Russet, Ute Russet, Centennial Russet, Wallowa Russet and Dakota Russet. ‘Russet Burbank’, the oldest Russet potato is also placed in this group and is closely clustered with breeding selection A06968-4. One of the prominent clones A06021-1T (to be released as ‘La Belle Russet’) is also placed in this group. *S*. *etuberosum* introgressed clones ETB6-21-3 and A00ETB12-2 are placed together in this group and are closely clustered with ‘Pallida CPC’, P2-4, ‘Snowden’ and ‘Chipeta’. In addition to *S*. *etuberosum*, this group includes prominent selections with potato virus Y and *Globodera pallida* resistance. Important clones in the pedigree include: ‘GemStar Russet’, ‘Gem Russet’, ‘Katahdin’, ‘Lenape’ and ‘Wischip’.

Group 1B comprised of 35 clones: 13 from NWPVD, nine from ME, five from CO, four from WI, two from ME and one each from NY and Agriculture Canada (Fig 2 and S2 File). Russet varieties clustered in this group include, Allagash Russet, Classic Russet, Clearwater Russet, Echo Russet, GemStar Russet Keystone Russet, Mesa Russet, Owyhee Russet, Silverton Russet and Teton Russet. ‘Mesa Russet’ a progeny of ‘Silverton Russet’ and ‘Teton Russet’ a progeny of ‘Classic Russet’ are placed in this group. In addition, two non-Russet clones ‘Shepody’ and Q115-6 (germplasm clone with tuber worm resistance) are also placed in this group. This group is also characterized by clones (Classic Russet, Owyhee Russet and Teton Russet) having tubers with typy appearance, an important trait for fresh market.

Group 1C is the smallest in this cluster with only five clones, four from NWPVD and one (AF4882-3) from ME (Fig 2 and S2 File). Major cultivar in this group is Ranger Russet, which is characterized by long tubers with good processing quality. AO96365-3, a selected progeny of ‘Ranger Russet’ with good processing traits is also placed in this group.

**Fig 3:**
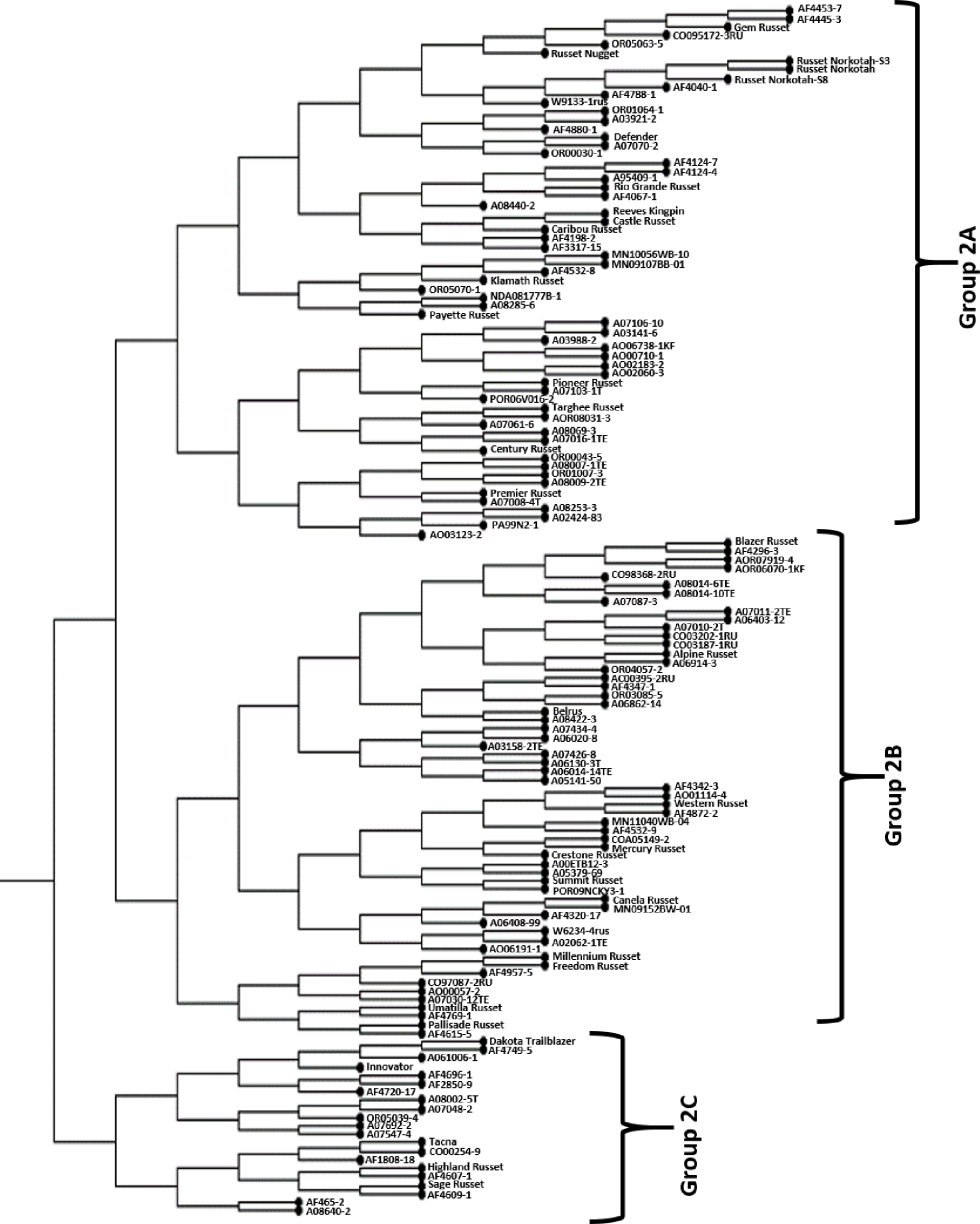
Detailed topology of Cluster 2.

Cluster 2 is the largest cluster with 142 clones. It is further divided into three groups, two major (2A and 2B) and one minor (2C) (Fig 3 and S3 File). Group 2A is the largest group with 63 clones, 41 from NWPVD, 13 from ME, five from CO, two from MN and one clone each from ND and WI. Similar to Group 1B, this group mainly consist of clones with fresh market potential namely, ‘Russet Norkotah’, ‘Reeves Kingpin’, ‘Rio Grande Russet’ and their offsprings. Out of 50 released Russet varieties, 14 clustered in this group: Castle Russet, Caribou Russet, Century Russet, Defender, Gem Russet, Klamath Russet, Payette Russet, Pioneer Russet, Premier Russet, Reeves Kingpin, Rio Grande Russet, Russet Norkotah, Russet Nugget, and Targhee Russet. ‘Russet Norkotah’ variant selections S3 and S8 are also placed in this group and are tightly clustered to ‘Russet Norkotah. ‘Caribou Russet’ and its female parent ‘Reeves Kingpin’, ‘Targhee Russet’ and its offspring, A07061-6 were placed next to each other in this group.

Group 2B composed of 59 clones, 35 from NWPVD, nine each from ME and CO, three from WI, two from MN and one clone from MD (Fig 3 and S3 File). Released Russet varieties in this group are Alpine Russet, Belrus, Blazer Russet, Canela Russet, Crestone Russet, Freedom Russet, Mercury Russet, Millennium Russet, Pallisade Russet, Summit Russet, Umatilla Russet and Western Russet. Surprisingly, *S*. *etuberousum* germplasm clone, A00ETB12-3 is also clustered in this group. Most of the released varieties (Blazer Russet, Mercury Russet, Pallisade Russet and Umatilla Russet) are clustered in this group with their offsprings.

Group 2C is composed of 21 clones, nine from NWPVD, eight from ME and one each from ND, CO, BV de ZPC, Netherlands and CIP (Fig 3 and S3 File). The prominent Russet varieties in this group include, Dakota Trailblazer, Highland Russet, Innovator, and Sage Russet. ‘Tacna’, a non-Russet variety released by CIP is also placed in this group.

**Fig 4:**
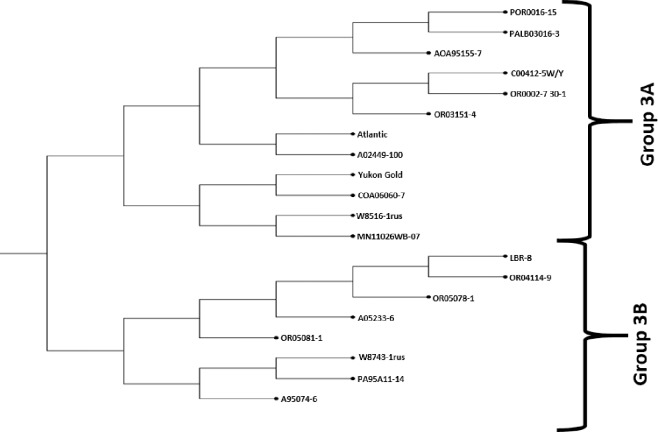
Detailed topology of Cluster 3.

Cluster 3 composed of 20 clones and is divided into two groups (3A and 3B) (Fig 4 and S4 File). Group 3A has 12 clones, six from NWPVD, two from CO and one clone each from MD, WI, MN and Agriculture Canada. Chip processing cultivar Atlantic and fresh market table stock specialty cultivar Yukon gold are placed in this group along with hybrids of ‘Russet Norkotah’. Group 3B consists of only eight clones, six from NWPVD and one each from CIP and WI. This is the smallest group and contains clones with late blight resistance from LBR-8 (S4 File).

Analysis of the grouping of clones in the clusters suggest that there is a thorough mixing of germplasm among the breeding programs. NWPVD clones that include clones from USDA-ARS potato-breeding program at Aberdeen, ID and potato-breeding and variety development program at Oregon State University, OR are evenly distributed in all the clusters. The USDA-ARS at Aberdeen, ID is a primary contributor for the NWPVD program with reciprocal exchange of material to other United States breeding programs. The results of the present study also support the fact that there is a continuous reciprocal exchange of breeding material among various potato-breeding programs, which has resulted in the clustering of mixed genotypes in the diversity analysis.

### Genetic structure analysis

Structure analysis was performed to determine the amount and distribution of genetic variation in Russet potato clones. Structure is an efficient software for examining genetic structure of different populations and infer the origins of individuals in an admixture population. Population structure of all the 264 clones used in this study was analyzed using a Bayesian-based approach in the admixture model. The evaluation of **Δ**K, using Evanno’s method showed a peak at K = 3 (S1 Fig), which indicated that the entire panel of 264 clones can be grouped into three clusters based upon the differences in their genetic makeup.

**Fig 5:**
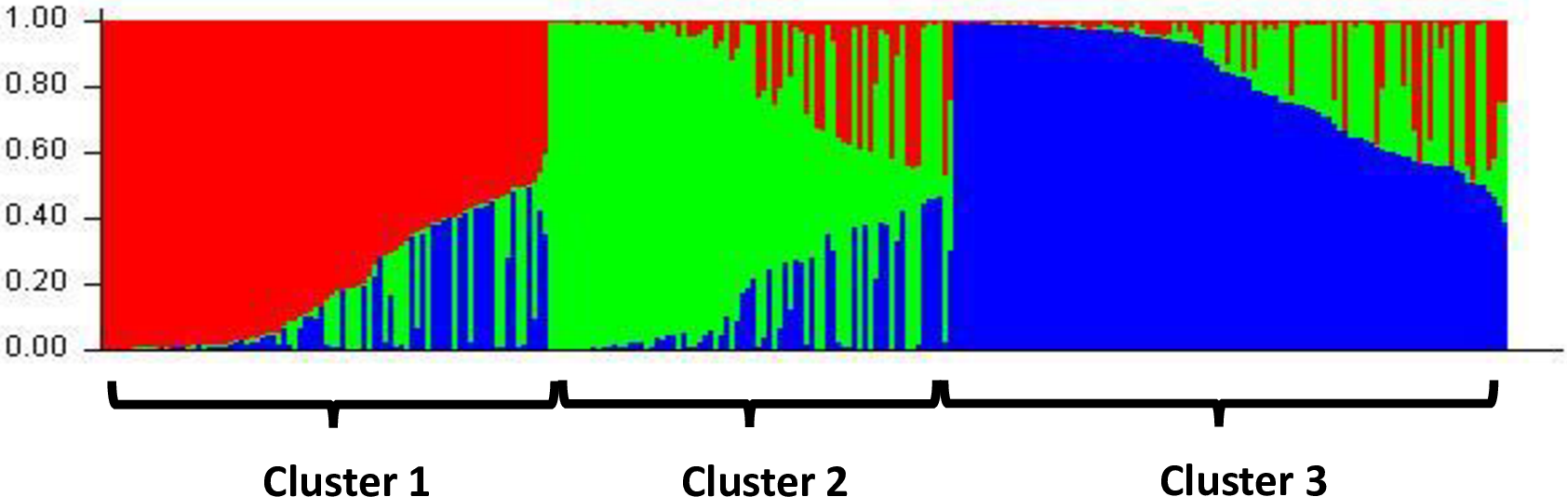
Structure analysis showing three distinct clusters of 264 Russet and non-Russet clones and varieties using 23 SSR primer pairs (Burnin = 100,000, MCMC = 200,000 with K = 3).

Structure analysis revealed significant admixture in the breeding material and no fixed clusters representing any particular group of clones based upon location of the breeding program were observed. There is one major (cluster 3) and two minor clusters (cluster1 and 2). All the genetic groups display a significant level of admixture present within each cluster (Fig 5 and S2 Fig). The detailed composition of three clusters with their pedigree and breeding programs is presented in the S5 File. Structure clusters show significant congruence with the clusters from the Neighbor Joining analysis; cluster 1, cluster 2 and cluster 3 roughly correspond to group 2, 1 and 3, respectively in NJ analysis. Cluster 3 is the largest among the three clusters with 104 clones, followed by cluster 1 and 2 with 84 and 76 clones respectively.

The most prominent released Russet varieties that grouped in cluster 1 include, Castle Russet, Dakota Trailblazer, Defender, Gem Russet, Mercury Russet, Premier Russet, Ranger Russet, Rio Grande Russet, Russet Burbank, Russet Norkotah, Russet Nugget and Western Russet. Three non-Russet clones: A00ETB12-3, Tacna and Yukon Nugget also grouped together in cluster 1.

The prominent Russet varieties in cluster 2 include, Allagash Russet, Caribou Russet, Coastal Russet, Centennial Russet, Freedom Russet, Gem Star, Payette Russet, Silverton Russet, Targhee Russet, Teton Russet and Ute Russet. Eight out of 16 non-Russet varieties used in the present study grouped in this cluster, which include Atlantic, Chipeta, LBR-8, Pallida CPC, P2-4, Red La Soda, Snowden and Q115-6 PTW.

Major Russet varieties in Cluster 3 include, Alpine Russet, Blazer Russet, Canela Russet, Century Russet, Clearwater Russet, Crestone Russet, Classic Russet, Dakota Russet, Echo Russet, Highland Russet, Klamath Russet, Keystone Russet, Mesa Russet, Millennium Russet, Owyhee Russet, Pallisade Russet, Reeves Kingpin, Sage Russet, Summit Russet, Umatilla Russet and Wallowa Russet. Non-Russet varieties that grouped in this cluster include Belrus, Innovator, Masquerade, Shepody and Yukon Gold.

In addition, breeding selection A06021-1T(to be released as La Bella Russet) was also placed in this cluster. The detailed summary of the entire three clusters with their pedigree and breeding program information is presented in S5 File.

Allele frequency divergence among all the three clusters ranged between 0.06 to 0.11 with a mean value of 0.08 that signifies moderate amount of gene flow between the sub-populations or sub-clusters (Table 4). This further, supports our presumption of continuous exchange of breeding material between various potato breeding programs. The average distance among the clones in the same cluster ranges from 0.39 to 0.44 with an average value of 0.41. Cluster 2 showed the highest heterozygosity among the individual clones, indicating it to be highly diverse whereas cluster 1 showed the lowest heterozygosity (Table 4). Fixation index (F_st_) measures the substructure and genetic diversity present in a set of individuals or subpopulations. In three of the clusters, F_st_ ranges from 0.25 to 0.29 with an average of 0.27, indicating significant differentiation among the 264 clones used in the present study.

**Table 4:** Allele frequency divergence (point estimates), average distances between individuals and mean fixation index (F_st_) of three clusters calculated using Structure 2.3.4.

Cluster No.	Allele-frequency divergence			Average distance between clones in the same cluster	Fixation index (F_st_)
	Cluster 1	Cluster 2	Cluster 3		
**Cluster 1**	-	0.11	0.06	0.39	0.29
**Cluster 2**	0.11	-	0.09	0.44	0.29
**Cluster 3**	0.06	0.09	-	0.40	0.25

### SSR markers as powerful fingerprinting tool

**Fig 6:**
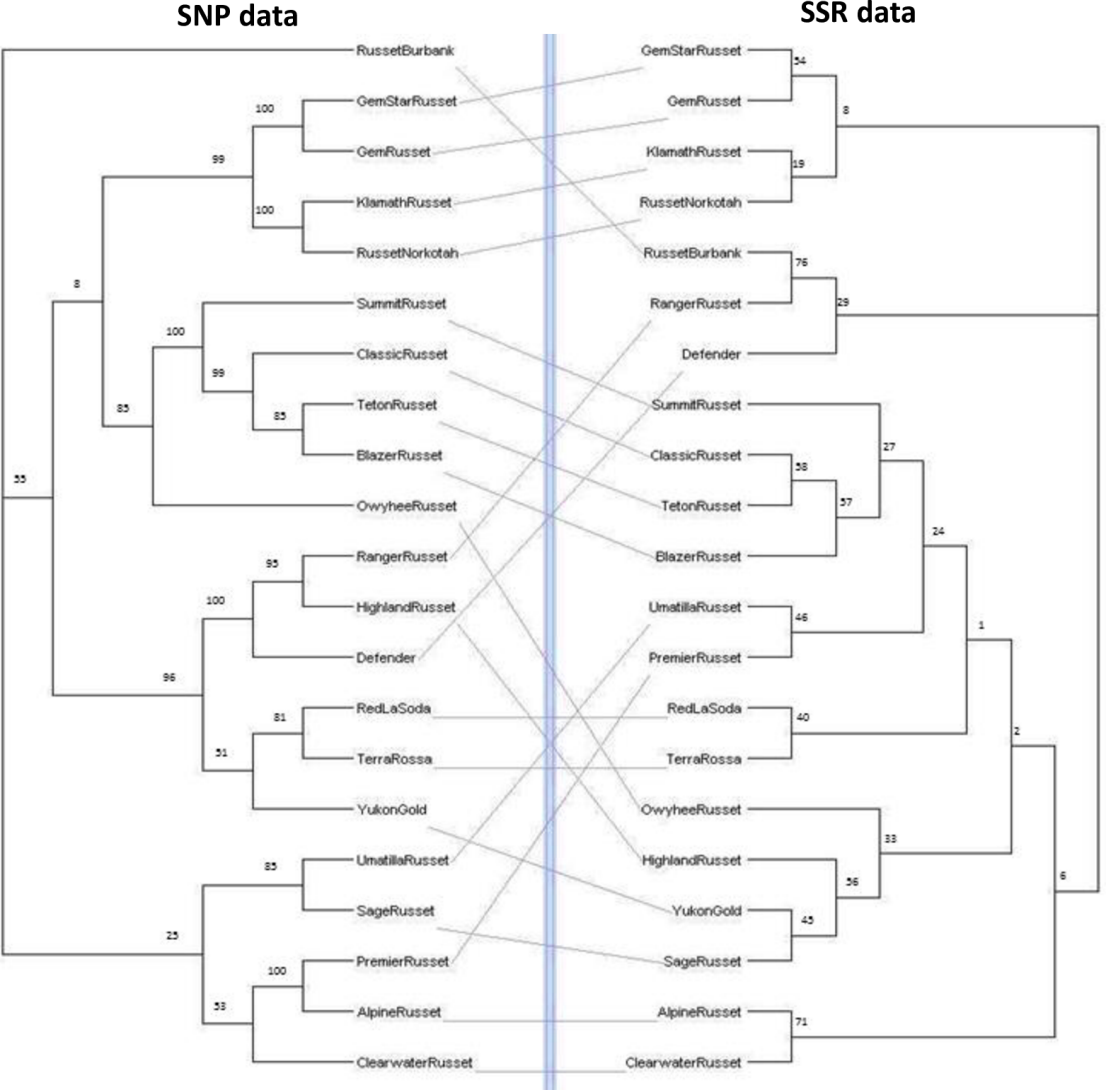
A tanglegram between the Neighbor Joining trees constructed using [1] 9979 SNP markers reported by Bali et al. 2017 and [2] 23 SSR markers generated in the present study.

SSR markers have the potential to detect high level of variation that increases the resolution for genetic diversity studies thus, reducing the number of markers required to distinguish between distinct genotypes. A subset of 21 clones (18 Russet and 3 specialty clones) was used to compare the power of SSR markers with SNP markers. The clustering analysis of these clones was performed using SNP data previously reported by Bali et al. [33] and SSR data generated in the present study. Comparison of clusters using tanglegram revealed that majority of the groupings show congruence between both the NJ trees (Fig 6). The SNP-based tree resulted into two clusters (one major and one minor) with ‘Russet Burbank’ as an outlier whereas the SSR marker-based NJ tree divided 21 clones into three clusters (one major and two minor (Fig 6). SSR data grouped ‘Russet Burbank’ along with ‘Ranger Russet’ and ‘Defender’ whereas SNP data presented it as an outlier. Defender, Highland Russet, Owyhee Russet, Premier Russet, Sage Russet, Umatilla Russet and Yukon Gold reshuffled in both the trees. SSR markers separate out Highland Russet, Owhyee Russet, Sage Russet and Yukon Gold into a different cluster. Overall, the grouping of clones is similar in both the analyses. Therefore, we propose that 23 informative SSRs can be as powerful as thousands of SNP markers to perform genetic diversity analysis in potato.

## Conclusion

In the present study, we are reporting the fingerprinting and diversity analysis of a large set of Russet breeding clones collected from various breeding programs across the United States. Our analysis could not separate Russet selections according to the breeding programs they originated from, which is an indicative of free-flow of germplasm among the potato breeding programs across the United States. Further, the SSR markers used in the study allowed the differentiation among Russet clones and varieties, and characterization of genetic relationships with the clustering of more closely related material. Characterization of genetic diversity of these clones can aid breeders in choosing desirable parents in breeding to exploit hybrid vigor and minimize inbreeding depression. Thus, these 23 SSR markers, separately or in tandem with SNP markers, can aid in variety identification, including misclassifications and duplications among varieties.

## Supporting information

S1 Table Summary of 198 Russet selections used in the present study (Northwest Potato Variety Development Program contains clones from Oregon State University; USDA/ARS, Aberdeen, Idaho). (DOCX)

S1 Fig The value of ΔK calculated using Evanno’s method K (1–10) after running STRUCTURE at 100,000 Burnin length and 200,000 MCMC reps. The presence of a peak at K = 3 depicted that 264 Russet and non-Russet clones could be divided into three clusters or groups.
(TIF)

S2 Fig Detailed description of genetic structure of 264 Russet and non-Russet clones inferred by STRUCTURE analysis run at 200,000 MCMC, 100,000 Burnin with K = 3. (TIF)

S1 File Genetic fingerprints of 264 potato clones generated using 23 simple sequence repeat (SSR) markers. (XLSX)

S2 File **Summary of all the clones (pedigree and breeding program information) present in Cluster 1 (Group A, B and C)**.
(XLSX)

S3 File **Summary of all the clones (pedigree and breeding program information) present in Cluster 2 (Group A, B and C)**.
(XLSX)

S4 File **Summary of all the clones (pedigree and breeding program information) present in Cluster 3 (Group A and B)**.
(XLSX)

S5 File Details of 264 Russet and non-Russet clones distributed in three clusters produced by STRUCTURE ran at Burnin = 100,000, MCMC = 200,000 with K = 3. (XLSX)
