# Supplementary material for: Evaluation of genetic diversity among Russet potato clones and varieties from breeding programs across the United States
Source: PLoS One. 2018 Aug 1;13(8):e0201415. doi: 10.1371/journal.pone.0201415 (PMC6070254; doi:10.1371/journal.pone.0201415)
Supplement: S1 Table — (DOCX) [file pone.0201415.s001.docx]

# S1 Table: Summary of 198 Russet selections used in the present study (Northwest Potato Variety Development Program contains clones from Oregon State University; USDA/ARS, Aberdeen, Idaho).

| **Breeding program** | **Sample Name** | **Female parent** | **Male parent** |
| --- | --- | --- | --- |
| Northwest Potato Variety Development Program (NWPVD) | A03158-2TE | A98292-2 | A98104-4 |
|  | A00324-1 | A95038-1 | GemStar Russet |
|  | A00ETB12-2 | A92303-7 | ETB6-21-3 |
|  | A02062-1TE | A97201-4 | A97299-1 |
|  | A02424-83LB | AO96781-4 | A97229-1 |
|  | A02449-100 | AO97011-1 | A97299-1 |
|  | A03141-6 | A98083-9 | A93157-6LS |
|  | A03921-2 | A96953-13 | A93005-10 |
|  | A03988-2 | A96783-55 | PA99N12-1 |
|  | A05141-50 | Rio Grande Russet | A93157-6LS |
|  | A05233-6LB | Palisade Russet | A00082-6 |
|  | A05379-69VR | A00ETB12-2 | Premier Russet |
|  | A05380-3 | A00ETB12-2 | A97214-4 |
|  | A06014-14TE | A98104-4 | COA00287-1 |
|  | A06020-8 | A99031-1TE | Premier Russet |
|  | A06021-1T | A99031-1TE | A96013-2 |
|  | A06029-4T | A99133-6 | A99034-2E |
|  | A06084-1TE | A98345-1 | A97267-1 |
|  | A06096-2 | A99007-12 | A98345-1 |
|  | A061006-1CR | PA03NM3-4 | Dakota Trailblazer |
|  | A061070-3CSR | PA03NM5-3 | Premier Russet |
|  | A06130-3T | Dakota Trailblazer | A99031-1TE |
|  | A06159-9 | Rio Grande Russet | Stampede Russet |
|  | A06176-4 | Canela Russet | A99034-2E |
|  | A06403-12 | A96783-109LB | PA03NM5-1 |
|  | A06408-99LB | A96814-65LB | A0094-1 |
|  | A06862-14VR | PA98V1-2 | A98345-1 |
|  | A06866-2PVY | PA98V1-2 | A00715-8 |
|  | A06914-3CR | A00715-8 | A93005-10 |
|  | A06968-4 | PA99N2-1 | A00731-2 |
|  | A07008-4T | A98082-17TE | A01025-4 |
|  | A07010-2T | A99006-2TE | A01054-4 |
|  | A07011-2TE | A99006-2TE | A02086-10 |
|  | A07016-1TE | A99007-2 | A02086-10 |
|  | A07030-12TE | Blazer Russet | A02086-10 |
|  | A07048-2 | A95409-1 | A01602-4 |
|  | A07061-6 | Targhee Russet | AOA95154-1 |
|  | A07070-2 | Targhee Russet | Premier Russet |
|  | A07087-3 | A02104-2 | A01025-4 |
|  | A07103-1T | Dakota Trailblazer | A01054-4 |
|  | A07106-10 | AOND95249-1Russ | A01025-4 |
|  | A07426-8LB | A98023-60LB | A01025-4 |
|  | A07434-4LB | A99375-41LB | A01602-4 |
|  | A07547-4VR | EGAO9702-2 | PALB0303-1 |
|  | A07692-2VR | A00681-4 | A02768-1 |
|  | A08002-5T | A96104-2 | AO96365-2 |
|  | A08007-1TE | A98374-1 | A02189-7 |
|  | A08009-2TE | A00727-1 | A02060-3TE |
|  | A08010-2TE | A01754-4 | A98196-5 |
|  | A08014-10TE | Blazer Russet | A98196-5 |
|  | A08014-6TE | Blazer Russet | A98196-5 |
|  | A08069-3 | Highland Russet | A98289-1 |
|  | A08253-3LB | A97070-51LB | AO96781-4LB |
|  | A08285-6 | A00477-103LB | AOND95249-1Russ |
|  | A08291-102 | A01235-33LB | A02093-1 |
|  | A08291-99LB | A01235-33LB | A02093-1 |
|  | A08311-2LB | Payette Russet | ND028673B-2Russ |
|  | A08422-3VR | A96953-13 | A02618-1 |
|  | A08433-4VR | A01667-3 | AOND95249-1Russ |
|  | A08440-2VR | A02611-1 | Premier Russet |
|  | A08640-2PCN | V15-71 | Rio Grande Russet |
|  | A91814-5 | NDA2031-2 | Ivory Crisp |
|  | A95074-6 | Agria | Summit Russet |
|  | A95409-1 | A89146-8 | Ranger Russet |
|  | A96104-2 | A88236-4 | A89512-3 |
|  | A98345-1 | Ranger Russet | Premier Russet |
|  | AO00057-2 | A91048-3 | A93116-3BSR |
|  | AO00710-1VR | A92030-5 | Liu |
|  | AO01114-4 | AO92017-6 | A86102-6 |
|  | AO02060-3 | A97201-4 | Premier Russet |
|  | AO02183-2 | A97236-3 | Premier Russet |
|  | AO03123-2 | A98082-17 | Premier Russet |
|  | AO06191-1 | A99134-1 | AC92009-4RU |
|  | AO06738-1KF | A96510-4Y | A92030-5 |
|  | AO06822-2 | AO95518-1 | Reiche |
|  | AO07469-2 | A01263-4LB | Stirling |
|  | AO96305-3 | A91018-6 | A89152-4 |
|  | AO96365-3 | A91141-1 | Ranger Russet |
|  | AO96781-4 | GemStar Russet | G6582-3 |
|  | AOA95155-7 | A81473-2 | A89163-3LS |
|  | AOR06070-1KF | Premier Russet | Sage Russet |
|  | AOR07919-4 | PA03NM3-4 | A00385-2 |
|  | AOR08031-3 | A01010-1 | Clearwater Russet |
|  | OR0002-7 30-1 | AO95506-3 | AC87084-3 |
|  | OR00030-1 | AO95495-7 | Russet Norkotah |
|  | OR00043-5 | AO95506-8 | Ranger Russet |
|  | OR01007-3 | PA98V2-1 | Yagana |
|  | OR01064-1 | AO95517-4 | Shepody / Yagana |
|  | OR03029-2 | AO96250-1 | AND9952-7 |
|  | OR03085-5 | PA99N82-4 | AO96250-1 |
|  | OR03151-4 | OR0002-6 | AO994110-203 |
|  | OR04057-2 | PA97B3-2 | AO994110-203 |
|  | OR04114-9LB | Amisk | LBR-8 |
|  | OR05039-4 | AO95245-2 | PA00N29-3 |
|  | OR05063-5 | OR0002-6 | Russet Norkotah Selection-3 |
|  | OR05070-1 | Russet Norkotah Selection-3 | Q174-2 |
|  | OR05075-1 | NY131 | AO94007-1 |
|  | OR05078-1 | NY131 | LBR-8 |
|  | OR05081-1 | PA99N82-4 | NY131 |
|  | OR08014-4 | PA99N12-1 | A00082-6 |
|  | OR09007-116 | PA99N82-4 | CO98067-7RU |
|  | OR09126-1 | CO98067-7RU | Canela Russet |
|  | OR09158-48 | OR00054-1 | GemStar Russet |
|  | OR09158-68 | OR00054-1 | GemStar Russet |
|  | ORAYT-9 | A88597-7 | A91048-3 |
|  | NDA081777B-1 | ND049589b-1Russ | Dakota Trailblazer |
|  | PALB03016-3 | P00LB5-3 | GemStar Russet |
|  | PA00N32-4 | PA95B2-66 | Summit Russet |
|  | PA95A11-14 | A8259-5 | A77715-6 |
|  | PA99N2-1 CRS | AO84275-3 | G6582-3 |
|  | POR0016-15VR | ? | ? |
|  | POR06V016-2 | PA01N55-3 | PA00N15-2 |
|  | POR09NCKY3-1 | PA99N82-4 | PA04LNC4-3Y |
|  | ETB6-21-3 | P2-3 | Katahdin |
| University of Maine, Orono, Maine (ME) | AF1552-5 | VW8303-3 | Shepody |
|  | AF1808-18 | AF1367-13 | OP |
|  | AF2199-6 | A84118-3 | AF295-10 |
|  | AF2596-2 | SC8805-12 | AF1811-6 |
|  | AF2850-9 | EB8309-3 | A8469-8 |
|  | AF3001-6 | Silverton Russet | AF1668-60 |
|  | AF3008-3 | AF1552-5 | A7816-14 |
|  | AF3016-2 | MN15620 | ARSW95-6527-1 |
|  | AF3317-15 | AWN86514-2 | Reeves Kingpin |
|  | AF4040-1 | LB9704-1 | Reeves Kingpin |
|  | AF4067-1 | 599275 | 98-3158-10 |
|  | AF4113-2 | Silverton Russet | A75188-3 |
|  | AF4124-4 | A8469-5 | SC9512-4 |
|  | AF4124-7 | A8469-5 | SC9512-4 |
|  | AF4172-2 | A95523-12 | A92158-3 |
|  | AF4191-2 | AO97044-17 | A95154-1 |
|  | AF4198-2 | A8469-5 | AF1753-16 |
|  | AF4281-3 | A98083-9 | A91814-5 |
|  | AF4283-1 | A98084-6 | A92030-5 |
|  | AF4296-3 | A0508-4 | A99081-8 |
|  | AF4320-17 | A99081-8 | A97214-4 |
|  | AF4320-7 | A99081-8 | A97214-4 |
|  | AF4342-3 | A97214-4 | A98295-3 |
|  | AF4347-1 | Ranger Russet | A98295-3 |
|  | AF4352-2 | Wallowa Russet | A98328-4 |
|  | AF4445-3 | Gem Russet | Defender |
|  | AF4453-7 | SA8312-1 | AF295-10 |
|  | AF4532-8 | ND4093-4 | CO82142-4 |
|  | AF4532-9 | ND4093-4 | CO82142-4 |
|  | AF4607-1 | Reeves Kingpin | Highland Russet |
|  | AF4609-1 | Reeves Kingpin | AO96164-1 |
|  | AF4615-5 | A97066-42LB | COA00329-3 |
|  | AF465-2 | 12-3 | 245-2 |
|  | AF4677-1 | A92030-5 | PALB03016-3 |
|  | AF4692-1 | A97070-51LB | Gem Russet |
|  | AF4696-1 | IND1072 | A00082-6 |
|  | AF4720-17 | A8469-5 | SC9010-4 |
|  | AF4749-5 | B5052-7 | A8469-5 |
|  | AF4769-1 | Gem Russet | Russet Norkotah |
|  | AF4788-1 | Reeves Kingpin | Russet Norkotah |
|  | AF4872-2 | Reeves Kingpin | Western Russet |
|  | AF4880-1 | AOND95249-1 | AND92475-2 |
|  | AF4882-3 | A85331-7 | COA00287-1 |
|  | AF4950-1 | W3160-5LB | W1151Rus |
|  | AF4950-2 | W3160-5LB | W1151Rus |
|  | AF4953-2 | W3160-51LB | W1836-3Rus |
|  | AF4953-6 | W3160-51LB | W1836-3 |
|  | AF4957-5 | W2683-2Rus | W1836-3Rus |
|  | AF4989-1 | ND03914AB-7Rus | ND039128B-2Rus |
| Colorado State University - San Luis Valley Research Center, Center, Colorado (CO) | CO00254-9 | AO95496-4 | NDTX4930-5W |
|  | CO03187-1RU | Rio Grande Russet | A9304-3 |
|  | CO03202-1RU | AC96010-3RU | Canela Russet |
|  | CO03276-4RU | CO95086-8RU | Blazer Russet |
|  | CO04220-7RU | CO96109-7RU | Summit Russet |
|  | CO05068-1RU | AWN86514-2 | CO98009-3RU |
|  | CO05175-1RU | Mesa Russet | AC96052-1RU |
|  | CO095172-3RU | Russet Nugget | AC88165-3 |
|  | CO95086-8RU | CO87009-4 | Silverton Russet |
|  | CO97087-2RU | CO87009-4 | W1005Rus |
|  | CO98067-7RU | Silverton Russet | TC1675-1 |
|  | CO98368-2RU | Russet Nugget | Bannock Russet |
|  | COA05149-2 | AC96052-1RU | Mercury Russet |
|  | COA06060-7 | Western Russet | Sage Ruset |
|  | C00412-5W/Y | German Butterball | TX1523-1RU/Y |
|  | AC00395-2RU | A95523-12 | Summit Russet |
| University of Minnesota, Minneapolis, Minneapolis (MN) | MN09075BW-01Rus | Single Hill Bulk | ?? |
|  | MN09107BB-01Rus | Single Hill Bulk | ?? |
|  | MN09135BW-01Rus | Single Hill Bulk | ?? |
|  | MN09152BW-01Rus | Single Hill Bulk | ?? |
|  | MN10053BW-01Rus | AC96052-1RU | CO98067-7RU |
|  | MN10054BW-01Rus | AC97306-1RU | Premier Russet |
|  | MN10056WB-10RUS | Summit Russet | Canela Russet |
|  | MN11026WB-07Rus | MN18710 | Russet Norkotah |
|  | MN11040WB-04Rus | MN02419 | Stampede Russet |
| University of Wisconsin, Madison, Wisconsin (WI) | W6234-4rus | Umatilla Russet | GemStar Russet |
|  | W8152-1rus | A93004-3 | Mesa Russet |
|  | W8516-1rus | Silverton Russet | Russet Norkotah |
|  | W8650-9 | WTS1269-3 | Dakota Pearl |
|  | W8743-1rus | AWN86514-2 | A91790-13 |
|  | W9133-1rus | ND4093-4 | CO82142-4RU |
|  | W9433-1rus | CalWhite | A96023-6 |
|  | W9492-4rus | Bannock | W1151rus |
|  | W9759-1rus | Blazer Russet | W3666-2rus |
|  | W9939-8rus | W6360-1rus | W2253-5rus |
