# Supplementary figures and images for: Evaluation of genetic diversity among Russet potato clones and varieties from breeding programs across the United States
Source: PLoS One. 2018 Aug 1;13(8):e0201415. doi: 10.1371/journal.pone.0201415 (PMC6070254; doi:10.1371/journal.pone.0201415)

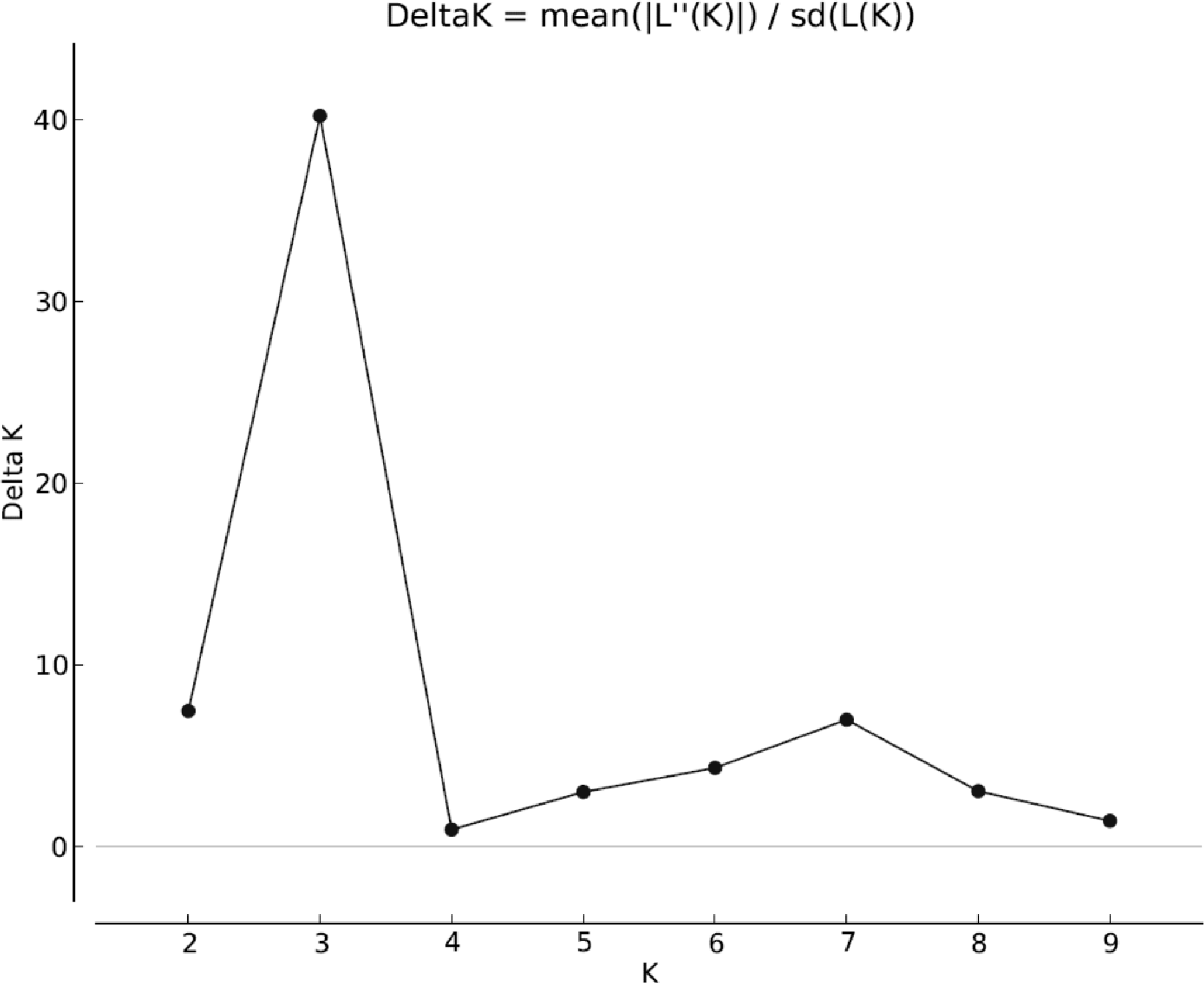

Supplement: S1 Fig — The presence of a peak at K = 3 depicted that 264 Russet and non-Russet clones could be divided into three clusters or groups. (TIF) [file pone.0201415.s002.tif]

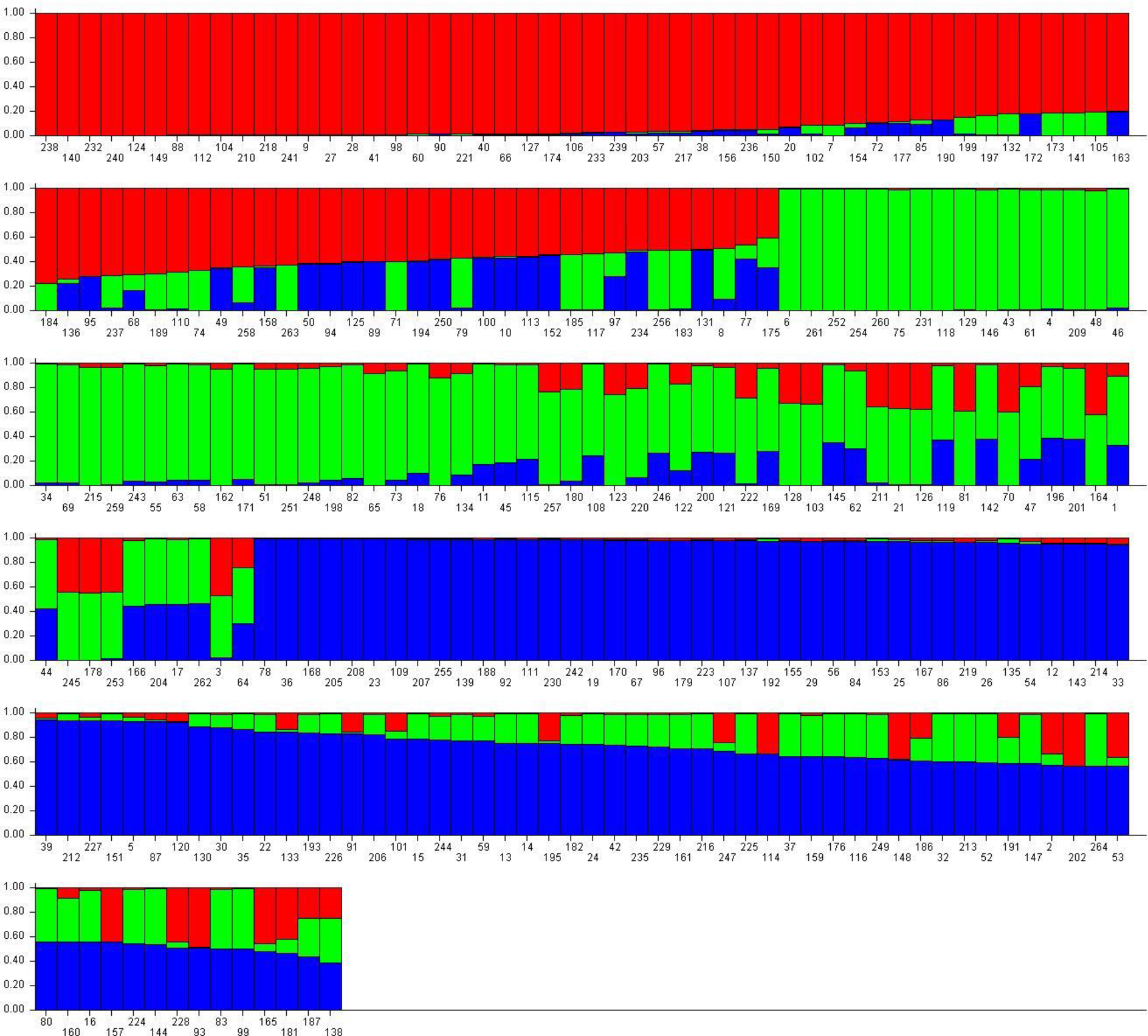

Supplement: S2 Fig — (TIF) [file pone.0201415.s003.tif]
